# Targeting of *Pseudomonas aeruginosa* cell surface via GP12, an *Escherichia coli* specific bacteriophage protein

**DOI:** 10.1038/s41598-021-04627-4

**Published:** 2022-01-14

**Authors:** George M. Ongwae, Mahendra D. Chordia, Jennie L. Cawley, Brianna E. Dalesandro, Nathan J. Wittenberg, Marcos M. Pires

**Affiliations:** 1 Department of Chemistry, University of Virginia, Charlottesville, VA 22904 USA; 2 Department of Chemistry, Lehigh University, Bethlehem, PA 18015 USA

**Keywords:** Pathogenesis, Viral proteins

## Abstract

Bacteriophages are highly abundant molecular machines that have evolved proteins to target the surface of host bacterial cells. Given the ubiquity of lipopolysaccharides (LPS) on the outer membrane of Gram-negative bacteria, we reasoned that targeting proteins from bacteriophages could be leveraged to target the surface of Gram-negative pathogens for biotechnological applications. To this end, a short tail fiber (GP12) from the T4 bacteriophage, which infects *Escherichia coli* (*E. coli*), was isolated and tested for the ability to adhere to whole bacterial cells. We found that, surprisingly, GP12 effectively bound the surface of *Pseudomonas aeruginosa* cells despite the established preferred host of T4 for *E. coli*. In efforts to elucidate why this binding pattern was observed, it was determined that the absence of the *O*-antigen region of LPS on *E. coli* improved cell surface tagging. This indicated that *O*-antigens play a significant role in controlling cell adhesion by T4. Probing GP12 and LPS interactions further using deletions of the enzymes involved in the biosynthetic pathway of LPS revealed the inner core oligosaccharide as a possible main target of GP12. Finally, we demonstrated the potential utility of GP12 for biomedical applications by showing that GP12-modified agarose beads resulted in the depletion of pathogenic bacteria from solution.

## Introduction

**Figure 1:**
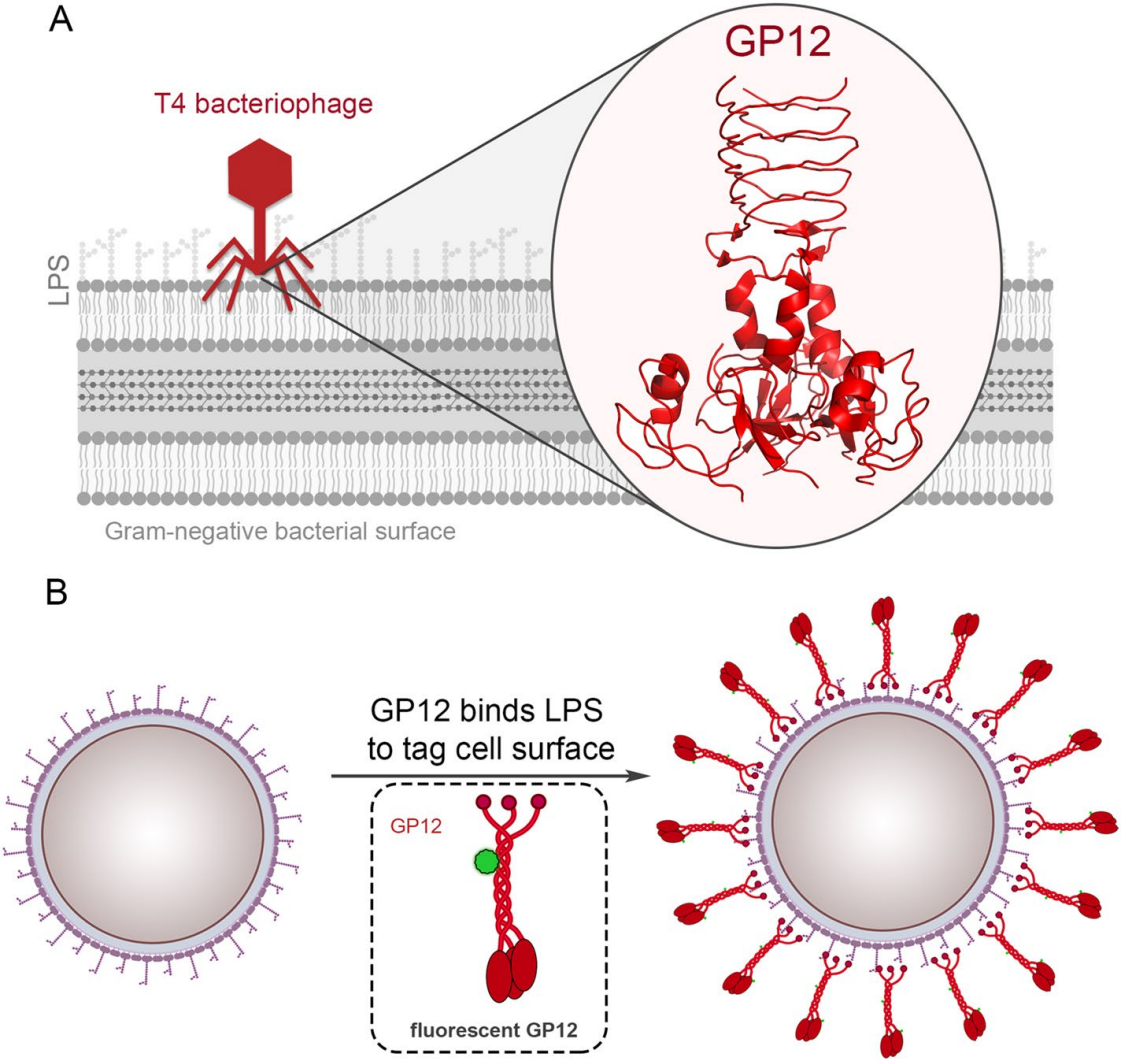
(**A**) Schematic representation of a T4 bacteriophage and its engagement with the LPS on the surface of its Gram-negative host cell. The short-tail fibers are responsible for surface adhesion during virus attachment, which are anchored at the base plate and bind to the core region of the host cell LPS. (**B**) The tagging of GP12 with a fluorescent handle is used to analyze binding of GP12 to the surface of a panel of bacteria, including Gram-negative pathogens.

Bacteriophages are the most abundant biological agents that exist in nature, reaching an estimated 10^31^ total viral particles^1,2^. Bacteriophage host specificity and mode of infectivity are features that rely primarily on the surface interactions between the viral particle and the host bacterial cells. Substantial differences in surface composition, particular variabilities in the specific structures that bacteriophages use to target bacterial hosts, can drive the type of engagements that occur between the two. Of all orders of viruses, tailed bacteriophages are the most common, of which the name arises from a flexible tail that is connected to an icosahedral head containing the viral genome (Fig. 1A). Within the class of tailed bacteriophages, T4 is a well-characterized virus that belongs to the family *Myoviridae* and it primarily infects the Gram-negative bacterium *Escherichia coli* (*E. coli*)^2^.

Bacteriophage T4 initiates the infection of *E. coli* by recognizing and binding to biomarkers on the cell surface^3^. One such prominent biomarker is the biomacromolecule lipopolysaccharide (LPS), which is a principal component of the outer membrane (OM) layer in a large number of Gram-negative bacteria. Bacteriophages often possess a number of receptor binding proteins (RBPs) that are displayed at the distal end of the tail and are involved in binding cell surfaces^4,5^. Among the RBPs found in T4 bacteriophages, a protein called GP12 (also known as a short tail fiber) was previously shown to have a unique affinity to LPS^5–8^.

The role of GP12 is to strongly adhere the T4 bacteriophage onto the surface of the host cells and to facilitate the injection of genetic material through the bacterial envelope by the bacteriophage. As such, binding of LPS by GP12 is extremely tight and has been previously described as pseudo-irreversible^5,7^. GP12 forms parallel homotrimers whereby the *C*-terminus end operates as an LPS-binding cavity. While T4 bacteriophages selectively infect *E. coli*, GP12 binds to a well-conserved portion of LPS that is found in many highly pathogenic Gram-negative bacteria. We envisioned that GP12 could, therefore, serve as a unique and highly specific mode of targeting surfaces of whole cells (Fig. 1B). Herein, we describe the binding pattern of GP12 across a number of Gram-negative bacteria. This is the first demonstration that isolated GP12 binds to the surface of potential bacterial pathogens. Unexpectedly, we observed a high level of binding by isolated GP12 towards *Pseudomonas aeruginosa* (*P. aeruginosa*). Moreover, when GP12 was covalently anchored onto agarose resin, it retained its ability to bind *P. aeruginosa* cells. We anticipate that GP12 may have utility in detection of *P. aeruginosa* due to its tight and preferential binding.

## Results and discussion

**Figure 2:**
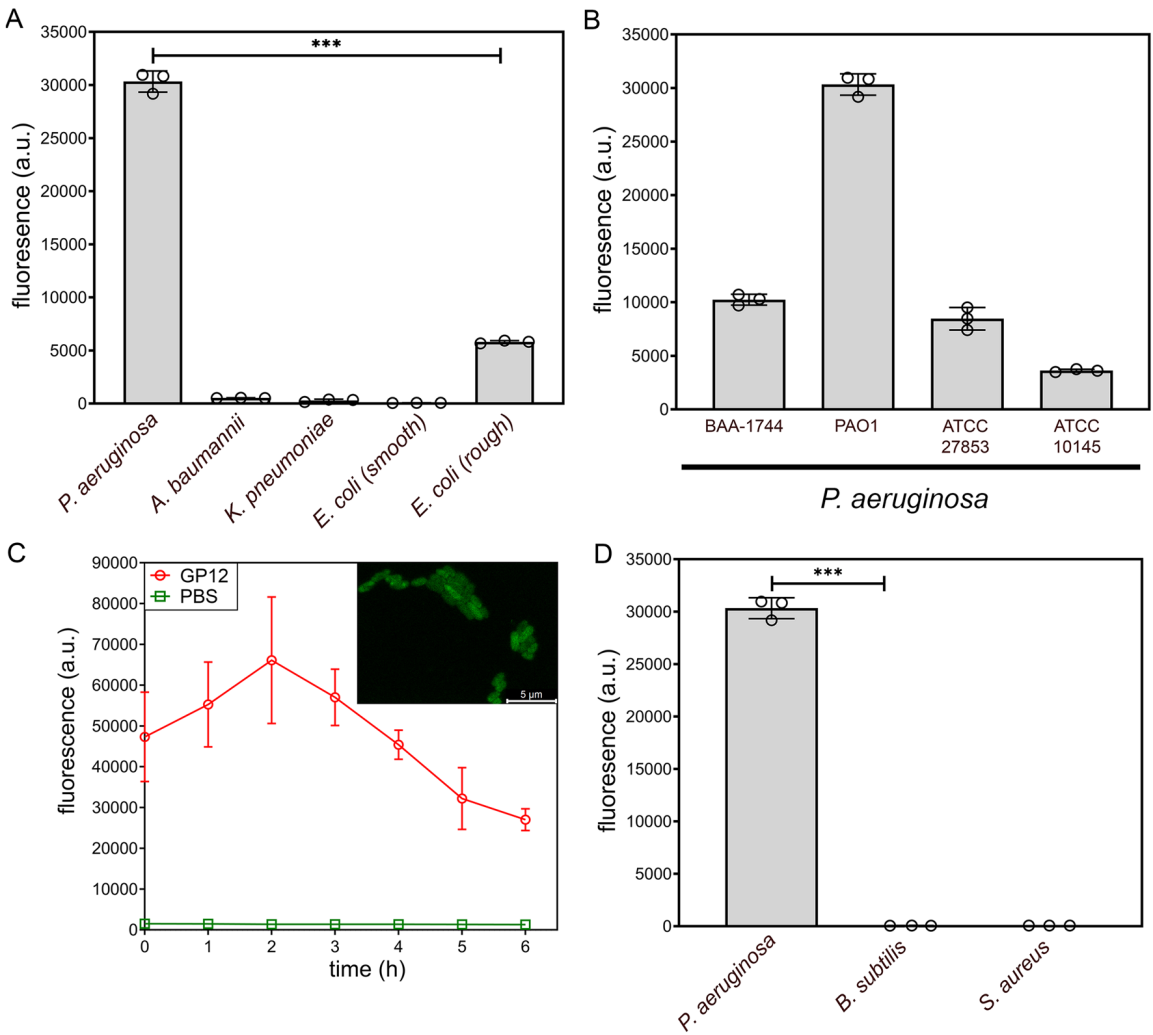
(**A**) Specified bacteria were incubated for 30 min with fluorescein-tagged GP12 (25 μM) and analyzed using flow cytometry. (**B**) Various strains of *P. aeruginosa* were incubated for 30 min with fluorescein-tagged GP12 and analyzed using flow cytometry. Please note that the levels for PAO1 were the same results measured in the screen of bacteria in panel (**A**). (**C**) *P. aeruginosa* (PAO1) was incubated with GP12, washed with PBS, and fluorescence was monitored over 6 h. *Inset*, fluorescence microscopy image of *P. aeruginosa* (PAO1) treated with fluorescein-tagged GP12. Data are represented as mean ± SD (n = 3). *p*-values were determined by a two-tailed *t*-test (*p < 0.05, **p < 0.01, ***p < 0.001, *ns* not significant). (**D**) Gram-positive bacteria and *P. aeruginosa* (PAO1) were incubated for 30 min with fluorescein-tagged GP12 and analyzed using flow cytometry.

First, GP12 was successfully expressed in *E. coli* and isolated to a high purity level in the absence of LPS (Fig. S1). GP12 has a key zinc ion that participates in LPS binding and precipitation^5^, so any associated LPS had to be released via a series of demetallations. The purified protein was then re-metallated and conjugated to fluorescein isothiocyanate (FITC) to introduce a fluorescent handle that could be used to track bacterial surface binding (Fig. 1B). Next, the association of the fluorescent GP12 to drug-sensitive strains of *Klebsiella pneumoniae* (*K. pneumoniae*), *Acinetobacter baumannii* (*A. baumannii*), *P. aeruginosa*, and *E. coli* was evaluated using flow cytometry (Fig. 2A). Surprisingly, high levels of surface labeling of *P. aeruginosa* was observed despite *E. coli* being the target host of T4 bacteriophages. In fact, labeling of *E. coli* was found to be at lower levels compared to *P. aeruginosa*. A similar experiment was performed with FITC-labeled Bovine Serum Albumin (BSA) and minimal cellular fluorescence was observed when two different strains of *P. aeruginosa* were treated with BSA-FITC (Fig. S1). While it is known that GP12 is important for the infectivity of *E. coli*, the infection machinery of T4 bacteriophages contain several other components aside from GP12, including other LPS binding proteins (e.g., long tail proteins) that can synergistically operate to select the host surface^9^. We anticipated that bacterial surface composition may be playing a role in the isolated GP12 binding. Therefore, we evaluated the surface tagging of rough *E. coli* strain, which lacks the *O*-antigen region of the LPS. Our data shows that comparatively, the presence of *O*-antigen appears to play a role in reducing labeling efficiency of GP12 to *E. coli*.

Interested in the high binding of GP12 to the *P. aeruginosa* surface, we sought to evaluate how various strains of *P. aeruginosa* may be susceptible to surface binding by GP12 (Fig. 2B). While there was some variability in labeling levels among the four *P. aeruginosa* strains tested, it was observed that they were all tagged by GP12. These results suggest that the LPS structure among these strains may possess conserved components that promote adhesion by GP12 in general. However, there may be some critical structural variability or accessibility detriment that is involved in inducing the range of binding levels observed. The tight association of GP12 for LPS could potentially be a significant advantage for future diagnostic and therapeutic applications. To evaluate the persistency of cell surface labeling, we tested the residency time of GP12 on the surface of *P. aeruginosa*. *P. aeruginosa* cells were incubated with GP12 to promote binding, unbound GP12 was washed away with PBS, and fluorescence levels were measured hourly for six hours in solution (Fig. 2C). Overall, cellular fluorescence levels remained unchanged for the first four hours, corresponding to no change in binding, after which there was a slow decrease in fluorescence, indicating a slight loss of binding. These results depict a high affinity of GP12 to its target in the context of a cellular surface which was further confirmed by fluorescence microscopy (Fig. 2C, inset). Two Gram-positive organisms, *Bacillus subtilis* (*B. subtilis*) and *Staphylococcus aureus* (*S. aureus*), were also tested using flow cytometry (Fig. 2D). As expected, due to the absence of LPS, labeling of both Gram-positive bacteria was found to be similar to background levels. These results suggest that GP12 binds to the Gram-negative cell surface in a LPS-dependent manner, since Gram-positive organisms lack LPS on their surface.

**Figure 3:**
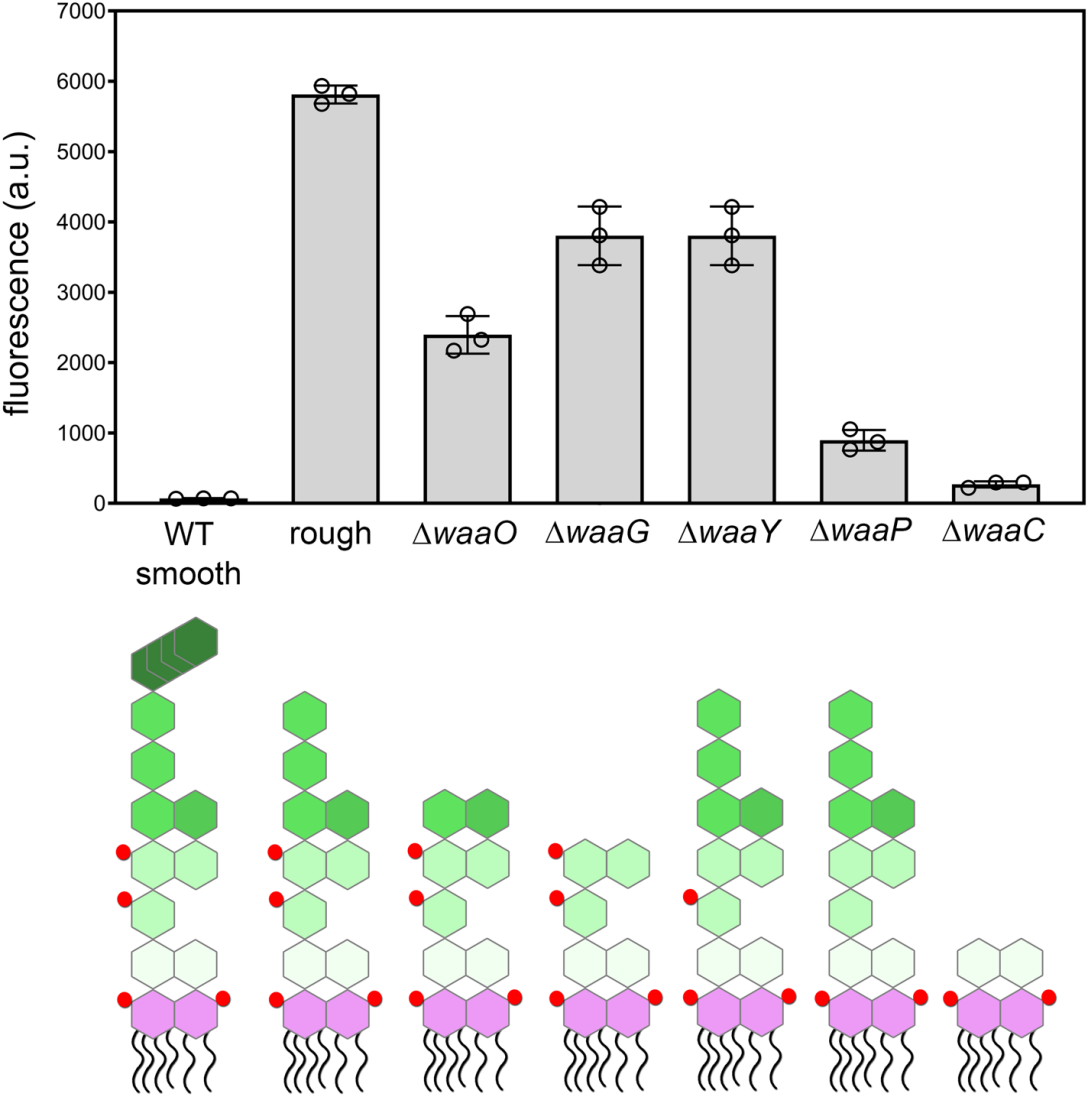
*E. coli* with various genetic deletions in the biosynthetic pathway of LPS were tested for their ability to bind fluorescently labeled GP12. Top, specified mutants were incubated for 30 min with fluorescein-tagged GP12 and analyzed using flow cytometry. Data are represented as mean ± SD (n = 3). Bottom, schematic representation of LPS that relates to the mutant shown in the top panel. The red spheres represent phosphorylation sites.

Next, we sought to gain further insight into the structural preferences of GP12 by using a series of genetic mutants in the biosynthetic pathway of LPS in *E. coli*. As observed earlier, the presence of *O*-antigen appears to disfavor binding of fluorescently labeled GP12 to the surface of *E. coli*. Similar to previous assays, cellular fluorescence levels were measured against a panel of *E. coli* strains with genetic knockouts in specific enzymes related to the core region of the LPS. In the ∆*waaO* mutant, a mutant that has the glucose unit removed, a decrease in cellular fluorescence was noted (Fig. 3). Further reduction of the terminal sugar units in the ∆*waaG* mutant reverted to higher labeling levels, as did the ∆*waaY* mutant, which lacks the WaaY kinase that is responsible for the phosphorylation of heptose. A significant decrease in GP12 binding was observed in *E. coli* lacking any heptose phosphorylation found in ∆*waaP*-knock out bacteria. Moreover, baseline fluorescence levels were observed in ∆*waaC*, which only display the KDO part of the inner core structure. Together, these results indicate that phosphorylation of the heptose in the inner core of the LPS and the core heptose units are necessary for proper association with free GP12.

**Figure 4:**
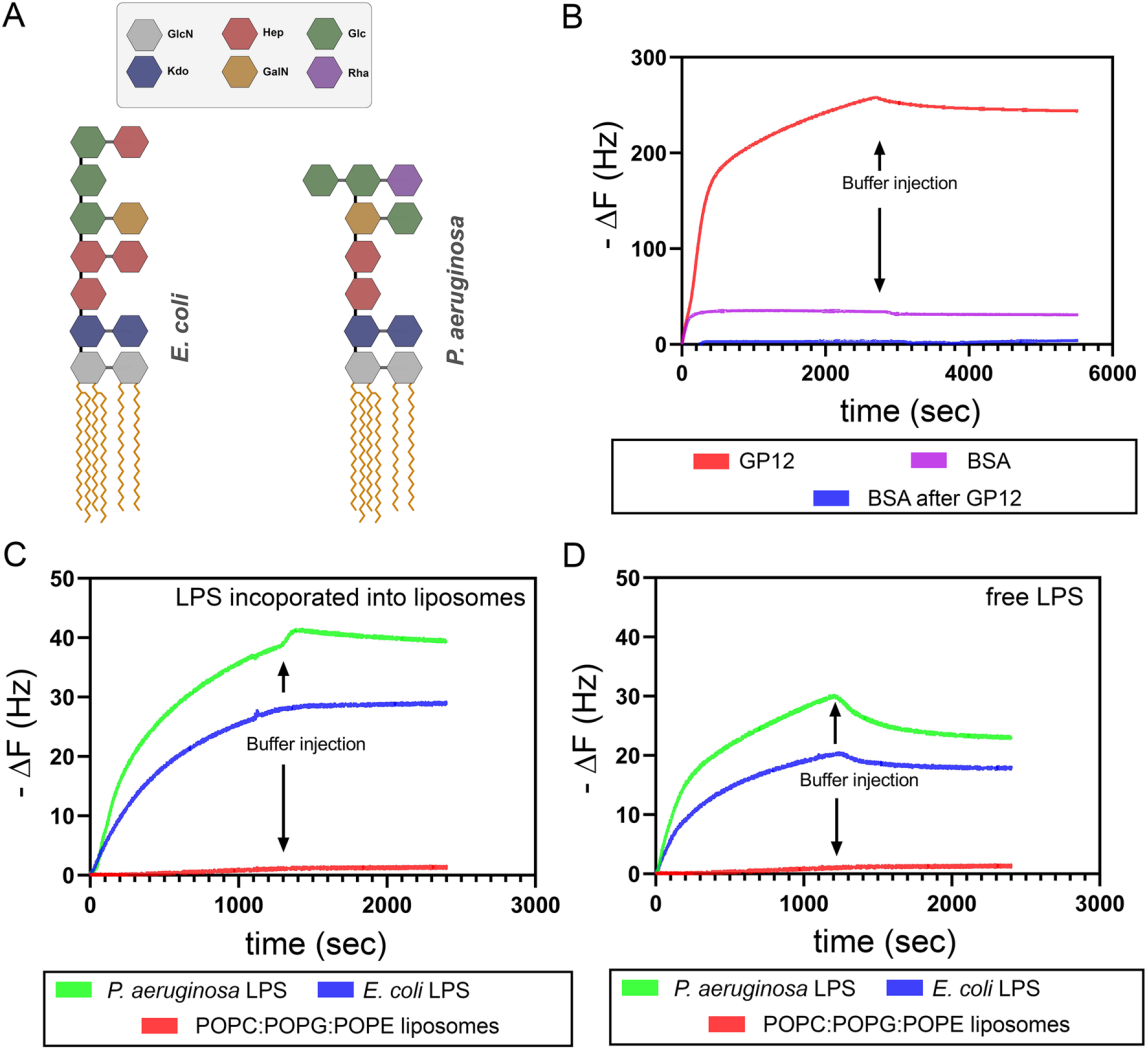
(**A**) Schematic representation of the lipid A and core oligosaccharide region of *E. coli* and *P. aeruginosa*. (**B**) QCM-D frequency shift (− ∆F) upon GP12 adsorption to gold coated sensor (red) and BSA adsorption on gold (purple). BSA injected after adsorption of GP12 minimally adsorbed to the surface (blue), indicating near complete GP12 surface coverage. (**C**) QCM-D frequency shift (− ∆F) as a function of time for liposomes binding GP12. Liposomes containing 20 wt.% *P. aeruginosa* LPS (green) or 20 wt.% *E. coli* LPS (blue) bind the GP12-coated surface, while LPS-free liposomes (red) do not bind. (**D**) QCM-D frequency shift (− ∆F) as a function of time for non-liposomal hydrated LPS and POPC:POPG:POPE liposomes binding to GP12. Suspensions of 0.25 mg mL^−1^ *P. aeruginosa* LPS (green) or *E. coli* LPS (blue) bind to the GP12-coated surface, while LPS-free liposomes (red) do not bind. All QCM-D binding studies were carried out in triplicate and representative binding curves are shown.

We reasoned that similarity in the LPS structure of *P. aeruginosa* and *E. coli* could account for the binding of GP12 to *P. aeruginosa* (Fig. 4A)^10^. The similarity is greatest in the lipid A and inner core regions. To further characterize the association of GP12 to its LPS target in both cell types, we performed a series of in vitro analyses. More specifically, we used the quartz crystal microbalance with dissipation monitoring (QCM-D) to examine the association of LPS to GP12. QCM-D is a surface-based sensing technique that relies on a piezoelectric quartz crystal that is coupled to an electric circuit^11^. A major advantage of QCM-D is that it enables label-free and real-time monitoring of biomolecules or particle adsorption, and it can be used to characterize interactions between an adsorbed receptor layer and particles, such as liposomes or viruses^12–14^. Adsorption of the receptor layer to the sensor and binding of liposomes to the receptor layer both result in mass accumulation on the sensor surface, which manifests as negative shifts in the resonant frequency of the quartz crystal. In these analyses, GP12 was first adsorbed onto a gold-coated sensor to function as a receptor layer for LPS binding (Fig. 4B). Our results showed that there was nearly complete coverage of GP12 onto the surface as demonstrated by the large shift in frequency upon exposure to GP12. To specifically evaluate the coverage, BSA was flowed after GP12 adsorption. The lack of BSA accumulation confirms that the entire surface was coated with GP12. As a control, BSA alone was adsorbed onto a bare gold-coated sensor and a clear shift in frequency was observed. Next, the binding of liposomes containing 20% of LPS from *P. aeruginosa* or 20% of LPS from *E. coli* was tested on the GP12-covered surface (Fig. 4C). A clear change in the frequency was observed upon the addition of liposomes containing both types of LPS. In fact, there was a larger frequency change with *P. aeruginosa* containing liposomes compared to *E. coli*. When LPS was not included in the preparation of the liposomes, there was no change in frequency observed. A similar pattern in frequency change was observed when the experiment was performed using free LPS (presumably in the micellular configuration) instead (Fig. 4D). Together, these results demonstrate that GP12 is capable of binding LPS from *P. aeruginosa* and it implies that the likely binding region within LPS is conserved between the two organisms.

**Figure 5:**
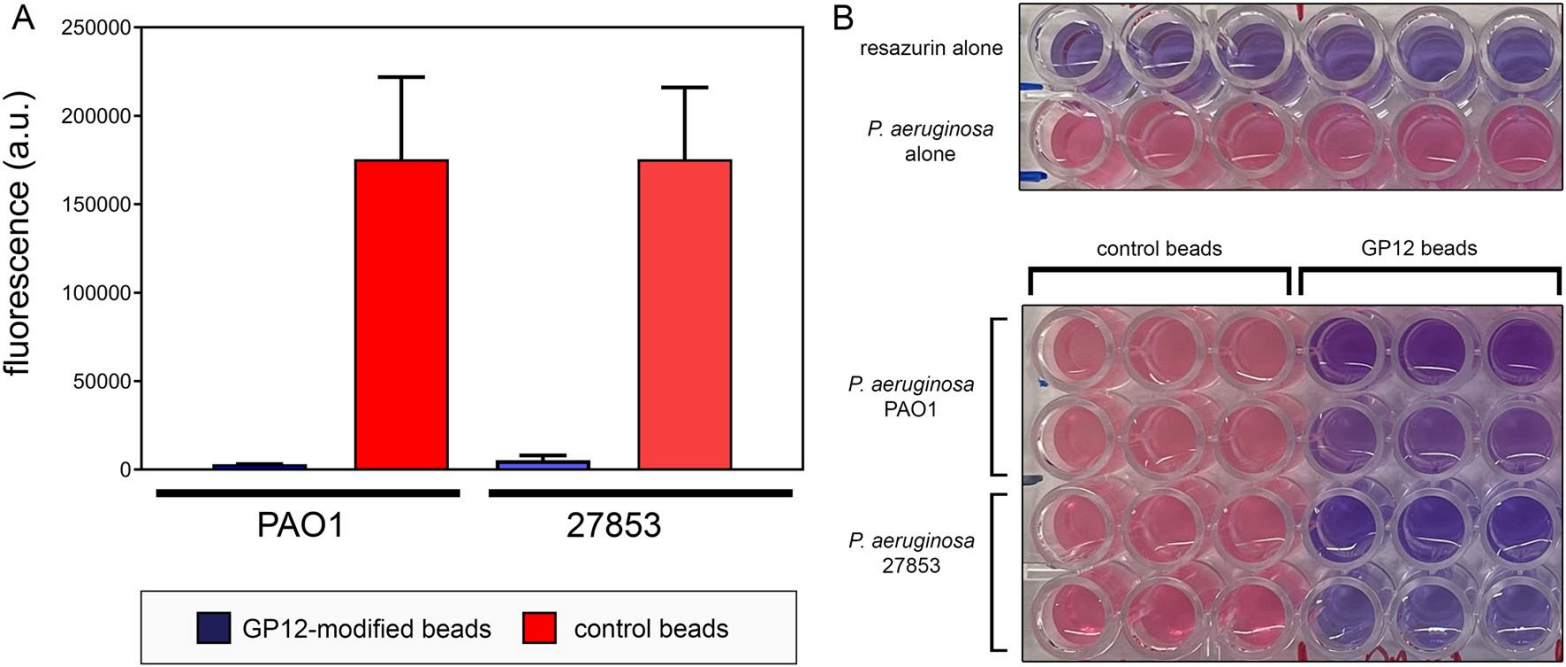
Binding of *P. aeruginosa* to agarose beads was assessed by measuring the fluorescence levels (**A**) and by visual inspection (**B**). *P. aeruginosa* was incubated for 60 min at 37 °C in the presence of agarose beads modified with GP12 or control beads in PBS. After this incubation period, the reagent resazurin was added and fluorescence was measured after an incubation time of 30 min. Fluorescence was measured by 550 nm excitation and 605 nm emission. Data are represented as mean ± SD (n = 3).

Finally, we decided to exploit the natural surface binding of GP12 for *P. aeruginosa* to establish the feasibility of assembling a pathogen capture material. Recently, there has been considerable efforts to engineer materials that enable fast diagnosis of bacterial pathogens based on their association to biocompatible surfaces^15,16^. Likewise, the adhesion of pathogenic bacteria to solid materials can potentially be the basis of extracorporeal devices that capture bacterial pathogens to mitigate blood infections^17–19^. The removal of bacterial cells from blood has the potential to improve the prognosis of septic patients. To this end, GP12 was covalently anchored onto agarose, a biocompatible material. The hydrophilicity of agarose beads along with their high surface area should lead to a high level of GP12 absorption and efficient enrichment of *P. aeruginosa* from solution. First, the presence of GP12 on agarose beads was confirmed using confocal microscopy (Fig. S2). To test the ability of GP12-modified beads to capture *P. aeruginosa* cells, the cells were incubated with unmodified control beads or GP12-modified beads (Fig. 5). After isolating the beads, a resazurin-based assay was performed on the remaining supernatant. The metabolic reduction of the oxidized blue dye by live bacterial cells causes a structural change that can be measured by the formation of a pink fluorophore. Our data showed a clear indication that GP12-modified beads, but not unmodified control beads, depleted live *P. aeruginosa* cells from solution as indicated by the lack of formation of a pink fluorophore. These results were confirmed by performing a colony forming units analysis (Fig. S3) and two different concentrations of the beads (Fig. S4). Moreover, we found that both strains of *P. aeruginosa* tested displayed similar adhesion to beads. Confocal microscopy confirmed the colocalization of GP12-modified agarose and bacterial cells (Figs. S5 and S6). Overall, these results establish that GP12 retains its ability to bind *P. aeruginosa* when anchored onto a solid material and will be further developed as a potential device to remove pathogenic bacteria from blood samples.

In conclusion, we have demonstrated that a tail fiber from the T4 bacteriophage displayed surprising propensity to bind *P. aeruginosa*. The binding of *P. aeruginosa* was observed in various strains including strains that are associated with difficult-to-treat infections in humans. Minimal binding was observed against the Gram-positive bacteria tested, which is consistent with LPS being the primary target of GP12. To further demonstrate the ability of GP12 to bind LPS from *P. aeruginosa*, an in vitro QCM-D assay was performed. Additionally, we investigated the importance of *O*-antigen modifications in *E. coli*-GP12 binding via the use of knock-out strains for enzymes related to the core region. Finally, we showed that agarose-beads modified with GP12 retained the ability to capture *P. aeruginosa* and these beads were able to deplete pathogenic bacterial cells from solution. Together, we anticipate that our results will establish the potential of this bacteriophage protein to bind *P. aeruginosa* and we will further develop GP12 for targeted destruction of pathogenic bacteria and for the development of biosensing devices.

## Supplementary Information

Supplementary Information.
