# Supplementary material for: Targeting of Pseudomonas aeruginosa cell surface via GP12, an Escherichia coli specific bacteriophage protein
Source: Sci Rep. 2022 Jan 14;12:721. doi: 10.1038/s41598-021-04627-4 (PMC8760310; doi:10.1038/s41598-021-04627-4)
Supplement: Supplementary file 1 — Supplementary Information. [file 41598_2021_4627_MOESM1_ESM.docx]

**Targeting of *Pseudomonas aeruginosa* Cell Surface via GP12, an *Escherichia coli* Specific Bacteriophage Protein**

George M. Ongwae, Mahendra D. Chordia, Jennie Cawley, Brianna E. Dalesandro, Nathan Wittenberg, and Marcos M. Pires^1*s^

^1^Department of Chemistry

University of Virginia

Charlottesville, VA 22904, USA

^2^Department of Chemistry

Lehigh University

Bethlehem, Pennsylvania 18015, United States

* corresponding author: mpires@virginia.edu

**Materials and methods**

**Materials:** All chemicals and reagents were purchased from Sigma-Millipore and Chem-Impex and used without further puriﬁcation. Solvents were purchased from Fisher Scientiﬁc, USA. Fluorophores DAPI (Sigma, Cat# D9542-5mg, Lot# 079M4003V) and fluorescein isothiocyanate isomer I (Chem-Impex, cat# 00860, lot#001521-201602, 98% purity) were procured and used as is. Resazurin-sodium salt (Cat# 14322, Batch # 0549740-23 was obtained from Cayman Chemical). NHS-activated dry Agarose resin was bought from Pierce (Thermo, Cat#26197, Lot# V1308312) stored in a desiccator at 4^o^C prior to use. Amicon^®^ Ultra-4 centrifugal filter (Millipore, MWCO 30K) was used for protein concentration. All cell lines were purchased from ATCC except *E. coli* strains with deletions in the LPS biosynthesis (kind gift from Dr. Eric Brown’s laboratory at McMaster University).

**Instruments**. Protein fluorescence of GP12 protein and fluorescein-labeled GP12 protein was measured with Synergy H1 microplate reader (BioTek Instruments) for fluorescence excitation at 488nm and emission at 520nm. The resazurin/resorufin-assay fluorescence measurements were performed on Synergy H1 microplate reader for excitation at 550nm and emission at 605nm. Flow cytometry experiments were performed at Attune™ NxT Acoustic Focusing cytometer equipped with sidekick autosampler (Invitrogen). Fluorescence microscopy was performed on either tauSTED (Leica Microsystem) or Zeiss 880/980 multiphoton Airy scan microscopy system.

**GP12 expression, purification, and characterization.** GP12 deposited in the protein repository as short tail fiber protein GP12, UniProtKB code P10930 (FIB12_BPT4) was cloned into an expression vector as a recombinant protein with its helper protein, GP57 (a kind gift from Mark J van Raaij). For expression, plasmid pT4g57g12 in BL21(DE3)* cloned by Burda and Miller (1999) and graciously donated to our lab by Mark J. van Raaij was used.**^13^** GP12 expression, purification and crystallization was reported earlier by the van Raaij laboratory.**^14^** With slight modification of protocol to purify GP12 in soluble form, *E. coli* Lemo21(DE3) harboring plasmid pT4g57g12/Lemo21(DE3) was grown in 1L of high salt LB medium (Miller TC grade, Sigma) containing ampicillin (100μg/mL) and chloramphenicol (34μg/mL) at 37 ℃. 1 mM IPTG was added when the cell density reached OD_600_ = 1.0 and grown at 25 ºC for 5 hours after induction with IPTG. Cells were harvested by centrifuging at 3500 rpm for 10min (Sorvall Legend T Centrifuge with 75006434 Swinging Bucket Rotor), LB media was discarded, and cells were washed in 1X PBS.

For purification, cells were resuspended in 50mL of lysis buffer (50 mM NaH_2_PO_4_, pH 7.2, 0.1 M NaCl, 2 mM MgSO_4_) and disrupted by sonication. Under these conditions, GP12 remains bound to *E. coli* LPS and the protein will be bound to the membrane fragments upon lysis. The lysate was centrifuged at 13,000 rpm for 15min on an Avanti J – E Centrifuge (Beckman Coulter) and the insoluble fraction was saved. To extract GP12 from membrane fragments, the insoluble fraction was resuspended in Tris-EDTA buffer (TE buffer) which is 40mM Tris-HCl and 10mM EDTA at pH 8.0. The EDTA is used to chelate Zn^2+^ and partially unfold the protein to reconstitute it afterwards with Zn ions; partially unfolded GP12 does not bind LPS. The insoluble fraction containing GP12 was extracted three times with 20mL portions of TE buffer. The supernatants were concentrated using saturated (NH_4_)_2_SO_4_ solution added at 4℃ to a final concentration of 1.8 M (NH_4_)_2_SO_4_. The supernatant was centrifuged to remove precipitated material which was tested for GP12 by SDS-PAGE. The precipitate was suspended in TE buffer and trace impurities were isolated by gel filtration (**Figure S1-a**) on a Superdex^TM^ 200 PG column HILoad^TM^16/600 (GE) using TE buffer as mobile phase (flow rate 0.8mL/min, 0.15MPa maximum pressure) and AKTAprime plus (GE Healthcare) for the solvents pump. Purity was determined by SDS-PAGE (**Figure S1-b**) and desired fractions were pooled and concentrated using saturated ammonium sulfate and stored at 2.4mg/mL concentration and a temperature of 4 ℃ in 50 mM PBS at pH 7.2 doped with 100mM ZnSO_4_. For these protein expression conditions, using 8L of inoculation yielded 8-10mg of GP12. To calculate protein concentration, both the Bradford assay with bovine serum albumin as the standard and UV absorbance (280 nm) were used. The molar extinction coefficient for UV absorbance was determined to be 54,000 /M/cm^2^ using the Gill and von Hippel method. **^15^**

**GP12 characterization.** Molecular weight was confirmed using SDS-PAGE for reducing gel, non-reducing gel, boiled samples, and non-boiled samples. Non-boiled GP12 samples not treated with a reducing agent (without beta-mercaptoethanol, BME) appear as a homotrimer at ~168kDa, boiled, reduced gel samples appear at 56kDa (**Figure SI 1-b**).

**Binding to LPS using QCM-D frequency analysis.** To investigate LPS-binding robustness of isolated GP12, quartz crystal microbalance (QCM) was used to determine a mass change due to binding of GP12 to LPS: liposomes made from POPC, POPG and POPE in the ratio 4:1:5 were used as a control. Measurements were obtained using QCM-D (Q-Sense E1 Explorer, Biolin Scientific) utilizing a 5 MHz AT-cut quartz crystal. Data are presented from the 3rd overtone and displayed as negative resonance frequency shift (-∆F). A gold-coated QCM-D sensor was cleaned prior to use in RCA-1 solution (5:1:1 DI water: NH_4_OH:H_2_O_2_) at 70 °C. After cleaning, the sensor was soaked for 5 minutes in DI water, rinsed to remove residual cleaning solution, and blown dry with N_2_ gas. The sensor was then subjected to a 10-minute UV-ozone treatment (ProCleaner Plus, Bioforce Nanosciences). Buffer was flowed within 5 minutes after mounting the sensor in the instrument. For all experiments, temperature was held constant at 23.0 °C, and all solutions were flowed at 100 μL/min. The sensor was coated by flowing 6 µg/mL GP12 in PBS until reaching a final frequency of approximately -220 Hz. This step was followed by a PBS buffer rinse until the frequency was stable. BSA was then introduced to confirm full surface coverage of GP12. LPS interaction with GP12 was observed by flowing a LPS suspension, LPS-containing liposomes, or LPS-free liposomes (as control) over the immobilized GP12 at 100 μL/min for 20 min, followed by a 20 min PBS buffer rinse to remove unbound LPS or liposomes. Liposomes lacking LPS were a Gram-positive bacteria model consisting of 1-palmitoyl-2-oleoyl-glycero-3-phosphocholine (POPC): palmitoyl-2-oleoyl-glycero-3-phospho-1’-rac-glycerol (POPG): palmitoyl-2-oleoyl-glycero-3-phosphoethanolamine (POPE) (Avanti Polar Lipids, USA) in a molar ratio of 4:1:5. In LPS containing liposomes, LPS was incorporated at 20 wt % with background lipids POPC:POPG:POPE scaled consistently. LPS from *E.coli* O111:B4 and from *P. aeruginosa* (procured from ATCC, Manassas, VA) were suspended in PBS at 0.025 mg/mL. Liposomes were prepared by mixing lipids dissolved in chloroform in their desired weight percent ratios, the solvent was evaporated under vacuum. The appropriate volume of hydrated LPS was added to the dried lipid film to reach the desired weight %, then the samples were vortexed, bath sonicated for 10 min at room temperature, then extruded (Mini-Extruder, Avanti Polar Lipids) through a 100 nm pore filter (polycarbonate track etch membranes, Avanti Polar Lipids). Prior to flowing liposomes in the QCM-D chamber, they were diluted to 0.1 mg/mL with PBS.

**Labeling of protein with FITC.** FITC labeling was done using a published method with some modifications. Briefly, 500mL of GP12 at concentration 2 mg/mL was added into a 15mL spin column (10 kDa MWCO, Amicon Ultra 4, Millipore) and filled with labeling buffer (0.05M boric acid, 0.2M NaCl at pH 9.2). The protein was spun down at 5000g force on an Avanti J – E Centrifuge until final volume reduced to 500mL. Then, 40mL of a 5mg/mL stock of FITC, in DMSO, was added to the protein solution in a spin column wrapped in aluminum foil and incubated for 2 hours at room temperature. The protein was washed with four volumes of 1X PBS (4mL each) containing 100uM ZnSO_4_ until the flow through was clear no observable fluorescence. Labeled protein was stored at 4 ºC in 1X PBS containing 100μM ZnSO_4_; GP12 is a metallo-protein that requires a Zn^2+^ ion to stabilize the homotrimer at the C terminus.**^17^** Bovine Serum Albumin conjugated to FITC was performed in a similar manner.

**Assessment of labeling selectivity using fluorescence intensity.** To test selectivity for Gram-negative bacteria, a selected panel of bacterial species was grown overnight in lysogeny broth (LB) from a glycerol stock until the cells reached confluency. 1mL of bacterial culture diluted to an OD_600nm_ of 1.00 was pipetted into a centrifuge tube and pelleted on a tabletop centrifuge (Eppendorf 5415D Centrifuge) at 6000 rpm for 4 minutes. The pellet was washed 3X with 1X PBS and resuspended in 1000uL of PBS. Using a 96-Well microtiter plate (Thermo Scientific™), 50uL of bacteria culture was aliquoted into each of three wells making a triplicate test. Then, 150 uL of GP12-FITC was added for a final concentration of 25uM GP12-FITC. The plate was incubated for 30min at 37ºC based on the observation by Miernikiewicz and coworkers that GP12 forms complexes with LPS within a few minutes of incubation, and aggregate size stabilizes within 45min of mixing.^18,19^  The plate was then spun at 4000 rpm for 4min in a plate holder centrifuge (Sorvall Legend T Centrifuge with 75006434 Swinging Bucket Rotor), the supernatant was discarded and the leftover cell pellet was resuspended and washed 3X in 1X PBS. In the final step, the cells were fixed with 2% formaldehyde (200 uL) and fluorescence intensity per cell was determined using the BD FACS Canto II or ATUNE NxT flow cytometer; l_ex_/l_em_ = 488nm/515nm or Attune™ NxT Acoustic Focusing cytometer using same Ex/Em filter.

***P. aeruginosa* adherence to GP12-FITC bound agarose beads.** FITC-labeled GP12 (1.0mg/mL, 200μL) was diluted with 0.8 mL of NaHCO_3_ buffer (pH 9.0). In a 15 mL conical tube, 300 mg of agarose-NHS bids were weighed and suspended in 2.0 mL of sterilized DI water. To the suspension of agarose-NHS beads was added diluted GP12-FITC solution in NaHCO_3_ buffer (pH 9.0). The suspension was stirred for 4 hours at room temperature on a rotary mixer. The suspension was then centrifuged at 3500 rpm for 5 min to pellet the beads. The supernatant was discarded and the pelleted beads were resuspended in 2.0 mL DI water containing ammonium hydroxide solution (200mL, 3%) to remove all unreacted NHS on beads. The mixture was stirred on a rotary mixer for 1 hour and the resulting suspension was centrifuged at 3500 rpm for 5 min after which the supernatant was discarded. The beads were washed 3X with 2.0 mL PBS buffer (pH 7.4) and the beads obtained after the final wash were used for adherence of cells. *P. aeruginosa* cells (strains PAO1 and ATCC 27853) were grown in 5mL of LB media overnight, following which 2mL of the culture was removed and centrifuged. The cell pellet was washed with 1 mL PBS and resuspended in 2.0mL PBS buffer (pH 7.4). Serial dilutions of cell suspension to obtain cell densities at 10^-2^, 10^-3^, 10^-4^, and 10^-5^ were performed. Cells from the vial with 10^-5^ cell density were first treated with DAPI to label them. DAPI solution was added (20 mL of 1.0mg/mL DAPI in water) to the cell suspension in PBS and incubated for 1 h at room temperature. Cell pellet was obtained upon centrifugation, washed and resuspended in PBS buffer (pH 7.4) to use for incubation with FITC-GP12 agarose beads prepared above. Cell adherence to the suspension of GP12-FITC-bound agarose beads was tested by adding either 50mL, 100mL, or 200mL of cells to separate vials each containing 200mL of the beads in PBS buffer at pH 7.4; the vials were incubated at 37^o^C for 1h. The suspensions were removed from the incubator, allowed to settle, and cooled to room temperature for 30 minutes. The supernatants were removed with a pipette and the beads were gently washed with PBS buffer and resuspended in PBS buffer for microscope slide preparation. The beads were finally loaded on warm agarose media, cooled for 1 minute before covering them with a cover slip prior to microscopic evaluation.

**Cell adherence to GP12-agarose beads using resazurin/resorufin assay.** Two types of agarose beads were prepared:

a) To prepare control beads (CONH_2_), agarose-NHS was treated with ammonia by taking 300 mg beads, suspending in 2.0 mL NaHCO_3_ buffer (pH 9.0), cooling down to 0^o^C, and adding ammonia solution (20%, 200mL) to the suspension. The suspension was stirred on a rotary mixer for 4h after which it was carefully neutralized with dilute HCl (0.1M), centrifuged to pellet the beads, and discarding the supernatant. The beads were washed 3X with 3mL of distilled water following which the bead suspension was freeze-dried using a lyophilizer (Free zone, 4.5L, Labconco) to form dry agarose-CONH_2_ beads

b) To prepare GP12-agarose beads, 300mg of agarose-NHS beads as cold suspension were treated with 1.2mL of GP12 protein diluted to 0.1mg/mL in 2mL of NaHCO_3_ buffer (pH 9.0). The mixture was stirred for 14h and carefully quenched with 200mL of 20% ammonia solution. The suspension was further stirred for 2h and centrifuged to remove the supernatant. The pelleted beads were resuspended in 3mL of water and treated with dil. HCl (0.1M, 0.5mL) to neutralize excess ammonia solution. The beads were centrifuged to remove the supernatant and successively washed 3X with 3mL of distilled water. The GP12-agarose beads were freeze-dried as above to obtain dry powder, the beads were then used for further assays.

In two sets for control (C) and for GP12-agarose (G), three vials for each set were prepared (as C1, C2, and C3; G1, G2, and G3), and into each vial, 50mg of either control beads or GP12-agarose beads were weighed. The beads in each vial were suspended in 300mL of PBS (pH 7.4) and to the suspension, 200mL of cells (as a 10^-7^ dilution of *P. aeruginosa*, PAO1 or *P. aeruginosa*, ATCC 27853) were added. The vials were then incubated at 37^o^C for 1 h. The vials were removed from the incubator and the beads were allowed to settle at room temperature for 10 minutes. 100 mL of supernatant from each vial was carefully removed and placed into a 96-well plate in duplicate and into each well, 100 mL of resazurin solution (0.1 mg/mL. diluted to 25 mL as final concentration) was added to each well, and the well plate was monitored for development of fluorescence over time in a microplate reader.

**Supporting Figures**

1. **(b)**
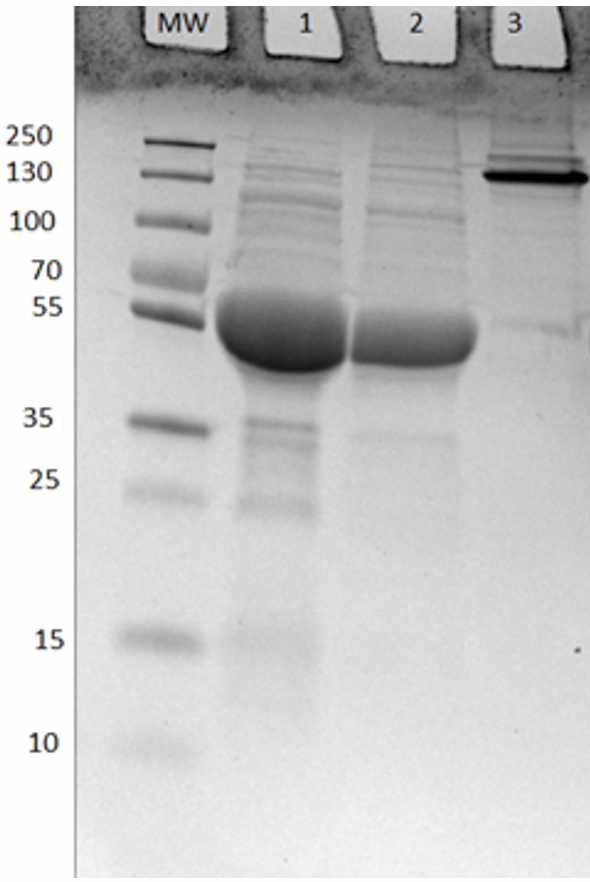


**Figure S1**: **(a)** FPLC chromatogram for purification of GP12 on a Superdex^TM^ 200 PG column with a pressure maximum of 0.15MPa at a flow rate of 0.8ml/min using TE buffer. The curve shows UV absorbance at 220nm with the prominent peak in the chromatogram confirmed to be GP12 by SDS-PAGE **(b)** Isolated GP12 SDS-PAGE analysis, the monomer has a MW of 56kDa and the homotrimer, 168kDa.From L-R: MW = molecular weight ladder; **1.** Gel filtration fraction, isolated peak ( +BME, +∆); **2.** Dilution of gel filtration fraction, isolated peak ( +BME, +∆); **3.** Dilution of gel filtration fraction, isolated peak ( −BME, −∆), [BME = betamercaptoethanol; ∆ = heat].

**
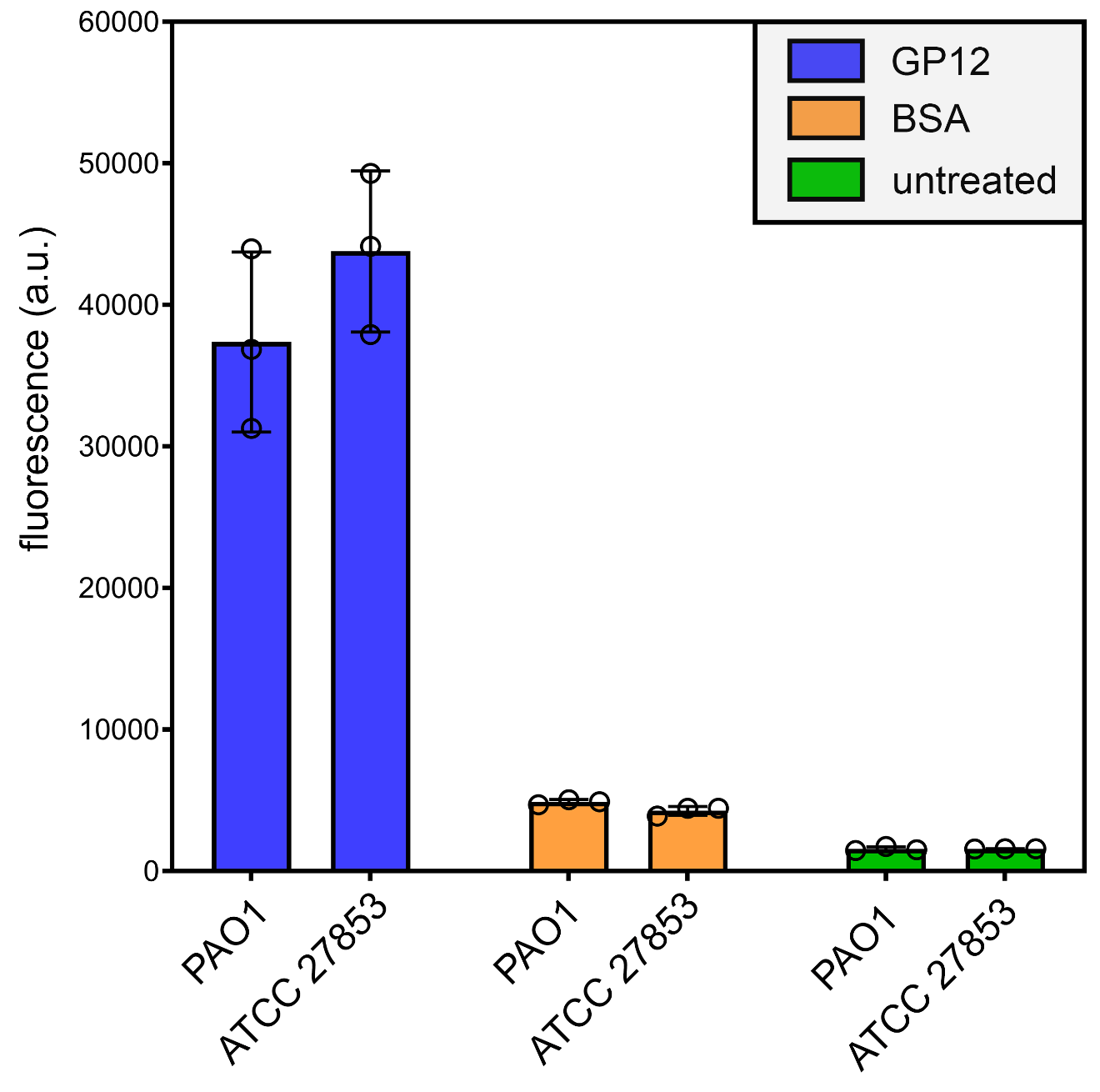
**

**Figure S2.** Specified bacteria were incubated for 30 min with fluorescein-tagged GP12 (25 μM), BSA (25 μM), or untreated and analyzed using flow cytometry. Data are represented as mean +/- SD (n = 3).

**
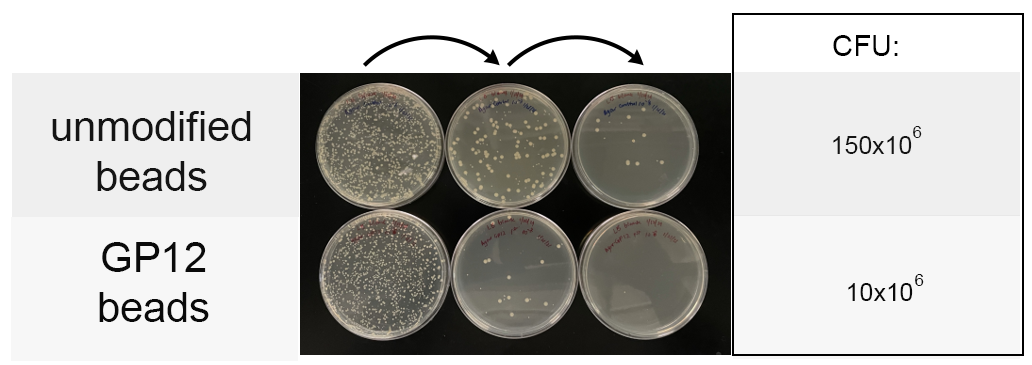
**

**Figure S3.** Colony forming unit assessment was performed in the media remaining after incubation with the designated beads in the presence of PAO1 (*P. aeruginosa*).

**
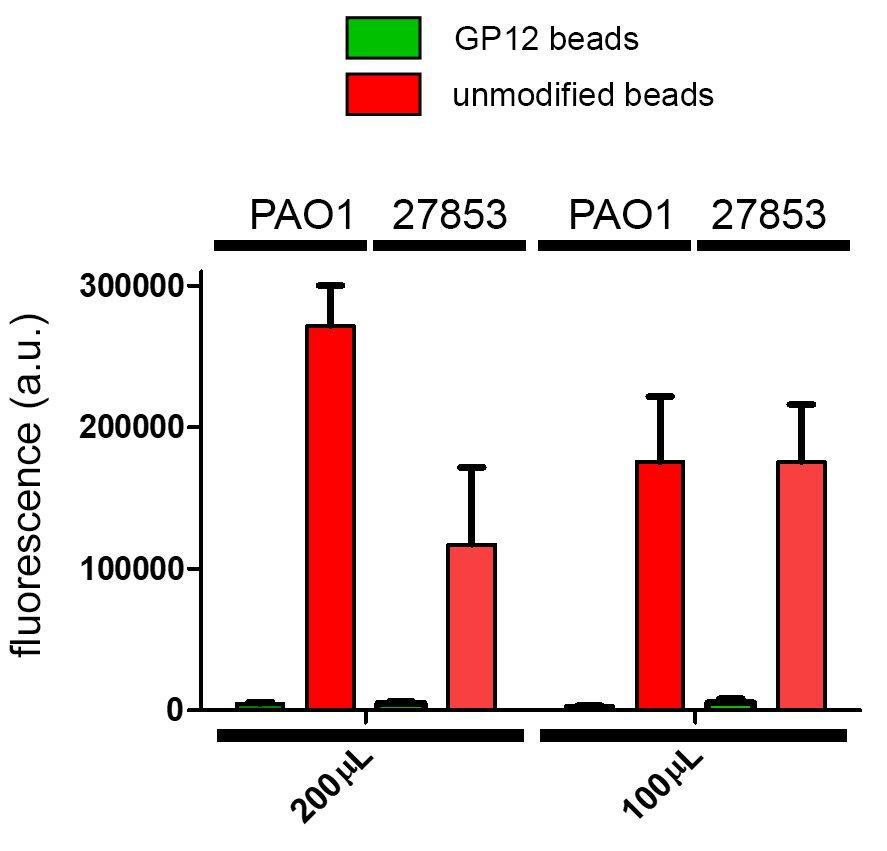
**

**Figure S4.** *P. aeruginosa* was incubated for 60 minutes at 37 ^o^C in the presence of agarose beads modified with GP12 or control beads (in two different volumes) in PBS. After this incubation period, the reagent resazurin was added and fluorescence was measured after an incubation time of 30 minutes. Fluorescence was measured by 550 nm excitation and 605 nm emission. Data are represented as mean +/- SD (n = 3).


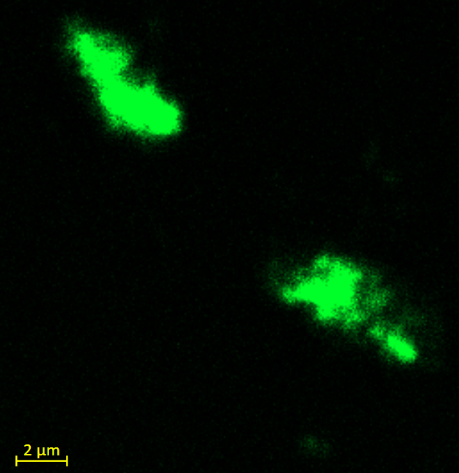


**Figure S5.** Confocal microscopy imaging analysis of NHS-agarose modified with GP12 tagged with fluorescein. Agarose beads were prepared by incubating fluorescein modified GP12 in the presence of NHS-activated agarose. After successive washing off of the free protein, protein-coated agarose beads were deposited on glass slides and imaged using tauSTED.


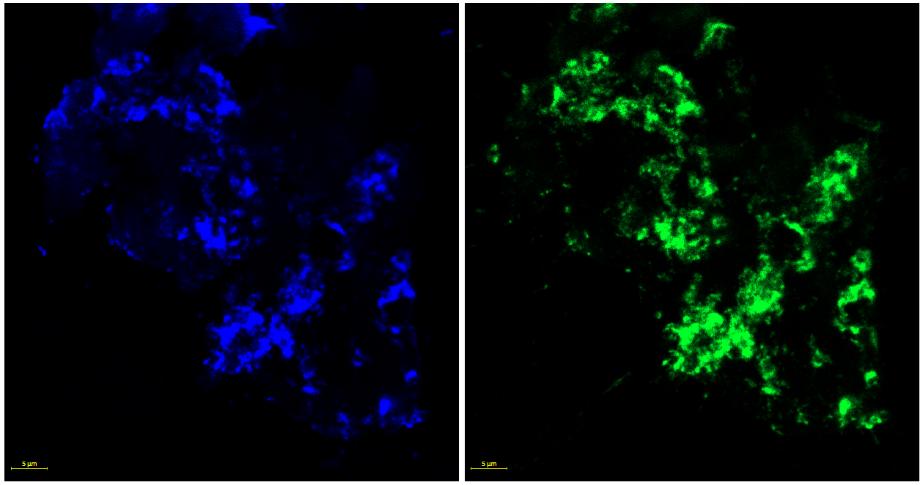


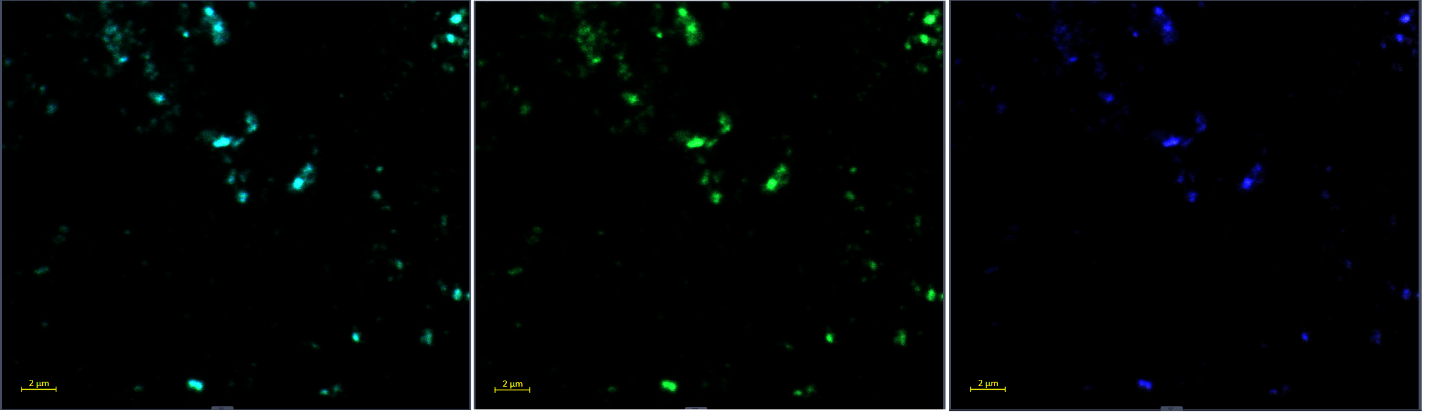


**Figure S6.** Confocal microscopy imaging analysis of NHS-agarose modified with GP12 tagged with fluorescein in the presence of *P. aeruginosa* (PAO1). GP12 modified agarose was incubated with *P. aeruginosa* (preincubated with DAPI) for 30 mins, beads were washed with PBS, and were deposited on glass slides and imaged using tauSTED.

Isolated gp12 PAGE (~57.5kDa for monomer and ~172.5kDA for homotrimer)


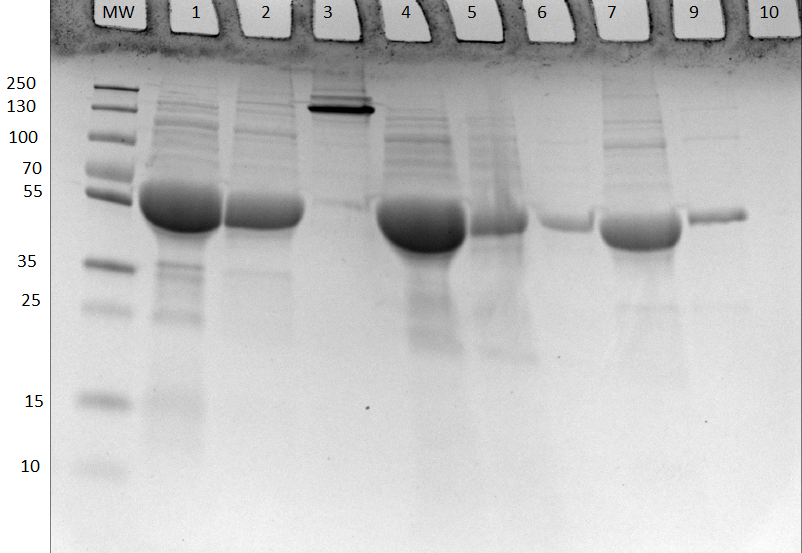


| MW | Thermofisher (Cat # 26619) | 5 | Run-1-Gel filtr. fraction, isolated peak ( +BME, +∆), 1:10 dil. |
| --- | --- | --- | --- |
| 1 | Run-2-Gel filtr. fraction, isolated peak ( +BME, +∆), 1:5 dili | 6 | Run-1-Gel filtr. fraction, isolated peak ( +BME, +∆), 1:20 dil. |
| 2 | Run-2-Gel filtr. fraction, isolated peak ( −BME, +∆), 1:5 dil | 7 | Run-3-Gel filtr. fraction, isolated peak ( +BME, +∆), 1:5 dil |
| 3 | Run-2-Gel filtr. fraction, isolated peak ( −BME, −∆), 1:5 dil | 8 | 1092uM gp12-FITC ( +BME, +∆), 1:5 dil |
| 4 | Run-1-Gel filtr.fraction, isolated peak ( +BME, +∆), 1:5 dil. | 9 | Blank (Running buffer) |

Table: Bacterial strains used in this study

| **Strain** | **Genotype** |
| --- | --- |
| *Pseudomonas aeruginosa* PAO1 |  |
| *Pseudomonas aeruginosa* ATCC 9721 |  |
| *Pseudomonas aeruginosa* BAA-1744 |  |
| *Pseudomonas aeruginosa* ATCC 27853 |  |
| *Pseudomonas aeruginosa ATCC 10145* |  |
| *Klebsiella penumoniae* ATCCC 13883 |  |
| *Acinetobacter baumannii* 3208 |  |
| *Escherichia coli* ATCC 25922 |  |
| *Escherichia coli* K-12 MG 1665 |  |
| *Escherichia coli* K-12 BW 25113 |  |
| *Escherichia coli* Lemo21(DE3) | Cm, Amp resistance |
| *Escherichia coli* DH5a Invitrogen Ref 18265-017 |  |
| *Escherichia coli* K-12 BW 25113 | Keio Collection DWaaG |
| *Escherichia coli* K-12 BW 25113 | Keio Collection DWaaO |
| *Escherichia coli* K-12 BW 25113 | Keio Collection DWaaY |
| *Escherichia coli* K-12 BW 25113 | Keio Collection DWaaP |
| *Escherichia coli* K-12 BW 25113 | Keio Collection DWaaC |
| *Escherichia coli* K-12 BW 25113 | Keio Collection DWaaL IS |
| *Bacillus subtilis* NCIB 3610 | genomic similarity to *B. subtilis* 168 |
| *Staphylococcus aureus* ATCC 26293 | Methicillin-sensitive |
| Staphylococcus aureus SeO1 | Methicillin-resistant |

**REFERENCES**

1. Hatfull, G. F., Dark Matter of the Biosphere: the Amazing World of Bacteriophage Diversity. *J Virol* **2015,** *89* (16), 8107-10.

2. Nobrega, F. L.; Vlot, M.; de Jonge, P. A.; Dreesens, L. L.; Beaumont, H. J. E.; Lavigne, R.; Dutilh, B. E.; Brouns, S. J. J., Targeting mechanisms of tailed bacteriophages. *Nat Rev Microbiol* **2018,** *16* (12), 760-773.

3. Hancock, R. E.; Reeves, P., Lipopolysaccharide-deficient, bacteriophage-resistant mutants of Escherichia coli K-12. *J Bacteriol* **1976,** *127* (1), 98-108.

4. Washizaki, A.; Yonesaki, T.; Otsuka, Y., Characterization of the interactions between Escherichia coli receptors, LPS and OmpC, and bacteriophage T4 long tail fibers. *Microbiologyopen* **2016,** *5* (6), 1003-1015.

5. Thomassen, E.; Gielen, G.; Schutz, M.; Schoehn, G.; Abrahams, J. P.; Miller, S.; van Raaij, M. J., The structure of the receptor-binding domain of the bacteriophage T4 short tail fibre reveals a knitted trimeric metal-binding fold. *J Mol Biol* **2003,** *331* (2), 361-73.

6. van Raaij, M. J.; Schoehn, G.; Burda, M. R.; Miller, S., Crystal structure of a heat and protease-stable part of the bacteriophage T4 short tail fibre. *J Mol Biol* **2001,** *314* (5), 1137-46.

7. Riede, I., Receptor specificity of the short tail fibres (gp12) of T-even type Escherichia coli phages. *Mol Gen Genet* ***1987*,** *206* (1), 110-5.

8. Raetz, C. R.; Reynolds, C. M.; Trent, M. S.; Bishop, R. E., Lipid A modification systems in gram-negative bacteria. *Annu Rev Biochem* **2007,** *76*, 295-329.

9. Zorzopulos, J. & Kozloff, L. M. (. Identiﬁcation of T4D bacteriophage gene product 12 as the baseplate zinc metalloprotein. *J. Biol. Chem.* ***1978***, 253, 5543–5547.

10. Thomassen E1, Gielen G, Schütz M, Schoehn G, Abrahams JP, Miller S, van Raaij MJ. The structure of the receptor-binding domain of the bacteriophage T4 short tail fibre reveals a knitted trimeric metal-binding fold. *J Mol Biol.* ***2003***, 331(2):361-73.

11. Kellenberger E, Stauffer E, Häner M, Lustig A, Karamata D. Mechanism of the long tail-fiber deployment of bacteriophages T-even and its role in adsorption, infection and sedimentation. *Biophys Chem.* ***1996***, 59(1-2):41‐59.

12. Lee, J., Patel, D. S., Stahle, J., Im, W. et al. CHARMM-GUI Membrane Builder for Complex Biological Membrane Simulations with Glycolipids and Lipoglycans. *J Chem Theory Comput.* ***2019***,15 (1), 775-786

13. Burda, M.R., Miller, S. Folding of coliphage T4 short tail fiber in vitro. Analysing the role of a bacteriophage-encoded chaperone. *Eur J Biochem*. *1999***,** 265(2):771-8

14. Thomassen E, Gielen G, Schütz M, Schoehn G, Abrahams JP, Miller S, van Raaij MJ. The structure of the receptor-binding domain of the bacteriophage T4 short tail fibre reveals a knitted trimeric metal-binding fold. *J Mol Biol.* ***2003***, 331(2):361-73.

15. Gill, S.C; von Hippel, B.H. Calculation of protein extinction coefficients from amino acid sequence data. *Analytical Biochemistry*. ***1989***, Volume 182, Issue 2, 319-326

1. Banks, P.R., Paquette, D.M. Comparison of three common amine reactive fluorescent probes used for conjugation to biomolecules by capillary zone electrophoresis. *Bioconjug Chem*. ***1995***, 6(4):447‐458.
2. Mason, W.S., Haselkorn, R. Product of T4 gene. ***1972***, *J. Mol. Biol.* 66, 445-469.
3. Miernikiewicz P, Kłopot A, Soluch R, Szkuta P, Kęska W, Hodyra-Stefaniak K, Konopka A, Nowak M, Lecion D, Kaźmierczak Z, Majewska J, Harhala M, Górski A, Dąbrowska K. T4 Phage Tail Adhesin Gp12 Counteracts LPS-Induced Inflammation In Vivo. *Front Microbiol*. ***2016***, 7:1112.
4. Isolde Riede, Klaus Drexler, Heinz Schwarz, Ulf Henning. T-even-type bacteriophages use an adhesin for recognition of cellular receptors. *Journal of Molecular Biology.* ***1987***, Volume 194, Issue 1,23-30.
